# Metabolic signature of obesity-associated insulin resistance and type 2 diabetes

**DOI:** 10.1186/s12967-019-2096-8

**Published:** 2019-10-22

**Authors:** Haya Al-Sulaiti, Ilhame Diboun, Maha V. Agha, Fatima F. S. Mohamed, Stephen Atkin, Alex S. Dömling, Mohamed A. Elrayess, Nayef A. Mazloum

**Affiliations:** 1 Department of Drug Design, University of Groningen, A. Deusinglaan 1, 9713 AV Groningen, The Netherlands; 2 Qatar Biomedical Research Institute (QBRI), Hamad Bin Khalifa University (HBKU), Doha, Qatar; 3 Weill Cornell Medicine-Qatar, Doha, Qatar; 4 Royal College of Surgeons, Ireland, Bahrain; 5 Biomedical Research Center (BRC), Qatar University, Doha, Qatar

**Keywords:** Metabolomics, Blood metabolites, Insulin sensitivity, Insulin resistance, Type 2 diabetes mellitus

## Abstract

**Background:**

Obesity is associated with an increased risk of insulin resistance and type 2 diabetes mellitus (T2DM). However, some obese individuals maintain their insulin sensitivity and exhibit a lower risk of associated comorbidities. The underlying metabolic pathways differentiating obese insulin sensitive (OIS) and obese insulin resistant (OIR) individuals remain unclear.

**Methods:**

In this study, 107 subjects underwent untargeted metabolomics of serum samples using the Metabolon platform. Thirty-two subjects were lean controls whilst 75 subjects were obese including 20 OIS, 41 OIR, and 14 T2DM individuals.

**Results:**

Our results showed that phospholipid metabolites including choline, glycerophosphoethanolamine and glycerophosphorylcholine were significantly altered from OIS when compared with OIR and T2DM individuals. Furthermore, our data confirmed changes in metabolic markers of liver disease, vascular disease and T2DM, such as 3-hydroxymyristate, dimethylarginine and 1,5-anhydroglucitol, respectively.

**Conclusion:**

This pilot data has identified phospholipid metabolites as potential novel biomarkers of obesity-associated insulin sensitivity and confirmed the association of known metabolites with increased risk of obesity-associated insulin resistance, with possible diagnostic and therapeutic applications. Further studies are warranted to confirm these associations in prospective cohorts and to investigate their functionality.

## Background

Obesity has become a global health care problem due to associated comorbidities including type 2 diabetes mellitus (T2DM), coronary artery disease (CAD), non-alcoholic fatty liver disease (NAFLD) and cancer [1–4]. However, a subset of obese individuals exhibit fewer comorbidities than their equally obese counterparts including maintaining their insulin sensitivity as well as having a healthier lipid profile [5]. The underlying protective mechanisms of the metabolically healthy obesity, also known as insulin sensitive obesity, remain unknown.

Previous studies have suggested that lower levels of inflammatory mediators play a role in the protective phenotype of obese insulin sensitive (OIS) individuals compared to their pathologically obese counterparts, also known as obese insulin resistant (OIR) individuals [6–8]. Other reports have suggested that OIS individuals show fewer markers of oxidative stress [8, 9]. These two mediators (inflammation and oxidative stress) could potentially be influenced by various genetic and environmental factors [10]. Although evidence of the genetic component remains limited, the environmental effect of certain pollutants and various medications has been previously established [11, 12].

Advancement in metabolomic tools including mass spectrometry (MS) technologies has allowed the identification of novel metabolic mediators of disease progression, including obesity associated insulin resistance and T2DM [13]. Recent evidence showed that adipose tissue from OIS, OIR and T2DM individuals exhibit a unique lipidomic signature associated with an increased risk of obesity-associated insulin resistance [14, 15]. Furthermore, metabolomics studies in individuals with T2DM have revealed several diabetes-associated metabolites, including 1,5-anhydroglucitol (1,5-AG), mannose and glucose [16, 17]. Additionally, lipidomics analysis of plasma samples from young adults has revealed that waist circumference was associated with levels of several sphingomyelins, diacylphosphatidylcholines and lysophosphatidylcholines, whereas HOMA-IR was associated with specific diacylphosphatidylcholines, lysophosphatidylcholines and diacylphosphatidylcholines [18]. However, no metabolomics studies have compared the metabolic differences in blood between lean healthy controls, OIS, OIR and T2DM. Such an approach can provide a deeper understanding of the underlying protective mechanisms in those lower risk individuals, and help in the design of novel diagnostic and therapeutic strategies targeting those at higher risk of disease [19, 20].

The aim of this study was to employ untargeted metabolomics analysis of blood samples from lean, OIS, OIR and obese-T2DM individuals in order to investigate the metabolic pathways underlying obesity-associated insulin resistance and T2DM.

## Methods

### Materials

Interleukin 6 (IL-6) and leptin ELISAs were from R&D systems (Abingdon, UK). Insulin ELISA was from Mercodia Diagnostics (Uppsala, Sweden). Other chemicals and reagents were from Sigma (Munich, Germany).

### Study design

One hundred and seven individuals (75 obese and 32 lean) were recruited at Al Emadi hospital and Hamad Medical Corporation. Lean participants were healthy females visiting the clinic for acne concerns. Obese participants were amongst patients undergoing weight reduction surgery. Subject inclusion criteria included males and females aged over 18 years and under 65 years of age. Subject exclusion criteria included malignancy or other terminal illness, poorly compliant patients, from whatever cause, inability to give informed consent, or involvement in other research projects. All individuals gave their written informed consent. Protocols were approved by Institutional Review Boards of the Anti-Doping Laboratory Qatar (X2017000224) and Weill Cornell Medicine-Qatar (15-00007). Measurements of body mass index (BMI), systolic blood pressure (SBP), diastolic blood pressure (DBP) and mean arterial blood pressure (MAP) were recorded. Fasting blood samples were obtained from all participants. Plasma cholesterol (total, HDL, LDL and triacylglycerol), fasting blood glucose (FBG) and liver function enzymes (total protein, ALP, AST, ALT and bilirubin) were measured by COBAS INTEGRA (Roche Diagnostics, Basil). IL-6, leptin and insulin were determined using commercially available ELISA. Insulin resistance was computed by homeostatic model assessment (HOMA-IR, https://www.dtu.ox.ac.uk/homacalculator/) [21] using 30th percentile (HOMA-IR = 2.4) as a threshold point. Accordingly, obese subjects (BMI > 30) were dichotomized into IS (HOMA-IR < 2.4, n = 20, 6 males and 14 females), IR (HOMA-IR > 2.4, n = 41, 15 males and 26 females) and 14 clinically diagnosed T2DM patients (9 males and 5 females) according to the definition of the American Diabetes Association (ADA) “Standards of Medical Care in Diabetes” [22].

### Metabolomics

Metabolomics profiling was performed using established protocols at Metabolon, Durham, NC, USA. All methods employed a Waters ACQUITY ultra-performance liquid chromatography (UPLC) and a Thermo Scientific Q-Exactive high resolution/accurate mass spectrometer interfaced with a heated electrospray ionization (HESI-II) source and Orbitrap mass analyzer operated at 35,000 mass resolution. The detailed description of the liquid chromatography-mass spectrometry (LC–MS) methodology was previously described [23, 24]. Briefly, serum samples from the 107 participants were methanol extracted to remove the protein fraction. The resulting extract was divided into five fractions: two for analysis by two separate reverse phase (RP)/UPLC-MS/MS methods with positive ion mode electrospray ionization (ESI), one for analysis by RP/UPLC-MS/MS with negative ion mode ESI, one for analysis by hydrophilic interaction chromatography (HILIC)/UPLC-MS/MS with negative ion mode ESI, and one sample was reserved for backup. Raw data was extracted, peak-identified, and quality control-processed using Metabolon’s hardware and software [25]. Compounds were identified by comparison to library entries of purified standards or recurrent unknown entities with more than 3300 commercially available purified standard compounds. Library matches for each compound were checked for each sample and corrected if necessary [26].

### Statistical analysis of metabolomics data

Statistical analyses were carried out using IBM SPSS version 25, R version 3.2.1 and SIMCA 13.0.1 software (Umetrics, Sweden). Variables with skewed distributions were log transformed or taken the square root of as appropriate to ensure normality [27]. Comparisons were performed with t-test, Wilcoxon–Mann–Whitney and 1-way ANOVA as appropriate. Significance was defined as p ≤ 0.05. Non-parametric tests were used for comparing ordinal or non-normal variables. Metabolomics data were log-transformed to ensure normality. Batch correction was performed by Metabolon by rescaling each metabolite’s median to 1. Principle component analysis (PCA) was performed using version 2.14, http://www.r-project.org/. PCA revealed two main components (PC1 and PC2) that together captured 27% of the variance in the data. Linear regression was performed to identify significant metabolites differentiating study groups (OIS vs OIR and T2DM) and (lean = 0, OIS = 1, OIR = 2, T2DM = 3, denoting disease progression) using the R statistical package (version 2.14, http://www.r-project.org/) after correcting for age, gender, BMI and principle components (PC1 and PC2). PCs represent common signals by the metabolites that contribute to the overall variance in the data and uncover fingerprints of confounders allowing their incorporation into the model by assigning them quantifiable measures. In the first model, the variable *study group* is categorical whereas the variable *disease progression* in the second group is continuous. Pathway enrichment analyses were carried out using Chi square tests to identify pathways with metabolites enriched at the top of the list of metabolites ranked by p-value from the linear model since Bonferroni level of significance was not observed. Orthogonal partial least square discriminant analysis (OPLS-DA) was used to compare lean, OIS, OIR and T2DM groups using SIMCA 14 with percentage of missing metabolite values across the samples of 50%. A partial correlation analysis was used to determine metabolic traits of disease (age, BMI, blood pressure, lipids, glucose/insulin/HOMA-IR and liver function enzymes) that exhibit best association with metabolites showing significantly differing levels between disease groups using IBM SPSS version 25, R version 3.2.1.

## Results

### General characteristics of participants

Thirty-two lean (BMI = 22.7 ± 2.5 kg/m^2^, all females) and seventy-five obese and morbidly obese (BMI = 45 ± 6.7 kg/m^2^, 45 females and 30 males) individuals were recruited at Hamad Medical Corporation and Al Emadi hospital, respectively. Lean individuals were younger and had significantly lower levels of SBP, MAP, triglycerides, triglycerides/HDL ratio, FBG, ALP, ALT and AST than obese individuals. Among obese participants, OIR individuals showed higher FBG than expected, suggesting a high prevalence of undiagnosed T2DM within this group. Therefore, subsequent analyses considered OIR and T2DM groups as one group (all IR) as both groups share obesity and insulin resistance. OIS subjects showed significantly lower MAP and levels of triacylglycerols, FBG, insulin and HOMA-IR than their equally obese all IR (OIR + T2DM) counterparts (Table 1).

**Table 1:** General characteristics of participants

Variables	Lean	OIS	OIR	T2DM	p value	All IR (OIR + T2DM)	p value
	(N = 32) (all F)	(N = 20) (4M + 16F)	(N = 41) (15M + 26F)	(N = 14) (9M + 5F)	ANOVA	(N = 55)	OIS vs (OIR + T2DM)
Age (years)	28 (6.8)	35.4 (10.0)	33.17 (10.1)	43 (10.9)	< 0.001	35.7 (1.49)	0.92
BMI (kg/m^2^)	22.7 (2.5)	45.7 (6.038)	45.2 (6.8)	43.3 (7.2)	< 0.001	44.8 (0.93)	0.55
SBP (mmHg)	115.3 (13.7)	124.9 (15)	126.9 (19.2)	132.3 (8.3)	0.004	128 (2.32)	0.42
DBP (mmHg)	70.7 (7.8)	74.2 (23.257)	74.0 (11.8)	77.1 (8.8)	0.52	74.8 (1.51)	0.88
MAP (mmHg)	85.6 (8.7)	85.2 (12.82)	91.7 (12.8)	95.8 (8.21)	0.01	92.7 (1.61)	0.03
Cholesterol (mmol/l)	4.3 (0.97)	4.5 (1.24)	4.8 (1.2)	4.9 (0.70)	0.27	4.8 (0.14)	0.24
LDL-cholesterol (mmol/l)	2.5 (0.96)	2.9 (0.89)	3.0 (1.05)	2.8 (0.66)	0.32	3.0 (0.13)	0.74
HDL-cholesterol (mmol/l)	1.4 (0.35)	1.2 (0.36)	1.4 (0.59)	1.2 (0.2)	0.18	1.4 (0.07)	0.12
Triacylglycerol (mmol/l)	0.8 (0.28)	1.1 (0.39)	1.3 (0.62)	1.8 (0.8)	< 0.001	1.4 (0.09)	0.04
Triglyceride/HDL	0.7 (0.56)	1.0 (0.45)	1.1 (0.77)	1.6 (1.1)	0.01	1.2 (0.12)	0.28
Leptin (ng/ml)	NA	60.2 (29.9)	51.2 (21.8)	38.9 (23.8)	0.05*	48.0 (3.09)	0.06
Adiponectin (μg/ml)	NA	4.2 (3.19)	3.1 (1.41)	3.4 (1.7)	0.5*	3.1 (0.30)	0.25
IL6 (pg/ml)	NA	3.7 (2.07)	4.3 (2.1)	4.0 (2.0)	0.45*	4.2 (0.27)	0.26
Insulin (pmol/l)	NA	5.3 (1.04)	6.3 (2.7)	11.3 (5.6)	< 0.001*	7.6 (0.57)	0.02
FBG (mmol/l)	5.0 (0.39)	6.3 (2.30)	17.9 (8.8)	15.1 (8.6)	< 0.001	17.2 (1.18)	< 0.001
HOMA-IR	NA	1.5 (0.55)	5.22 (3.2)	6.4 (3.0)	< 0.001*	5.5 (0.43)	< 0.001
TP (g/l)	73.7 (3.40)	70.3 (4.36)	71 (4.4)	74.3 (7.2)	0.04	71.8 (0.89)	0.34
ALP (U/l)	60.3 (17.1)	70.2 (18.38)	72.5 (16.1)	95.5 (38.1)	< 0.001	77.9 (3.46)	0.21
ALT (U/l)	12.6 (5.5)	22.9 (15.16)	31.3 (25.6)	30.9 (19.0)	0.002	31.2 (3.33)	0.15
AST (U/l)	15.5 (4.7)	20.8 (7.84)	24.9 (16.7)	21.7 (10.7)	0.04	24.1 (2.17)	0.36
Bilirubin (μmol/l)	21.2 (4.6)	8.30 (3.84)	8.2 (4.4)	8.5 (3.5)	0.55	8.0 (0.60)	0.9

*BMI* body mass index, *SBP* systolic blood pressure, *DBP* diastolic blood pressure, *MAP* mean arterial blood pressure, *LDL* low density lipoprotein, *HDL* high density lipoprotein, *IL-6* interleukin 6, *FBG* fasting blood glucose, *HOMA-IR* homeostatic model assessment of insulin resistance, *TP* total protein, *ALP* alkaline phosphatase, *ALT* alanine transaminase, *AST* aspartate aminotransferase, *F* female, *M* male. Data are presented as mean (SD). Differences between OIS, OIR and T2DM were tested by ANOVA. Differences between (OIS and OIR) and (OIS vs OIR + T2DM) were tested by independent sample t test (normally distributed variables) or Mann–Whitney U (variables with skewed distribution) test. A p-value significance level of 0.05 was used. The asterisk (*) denotes ANOVA that compared OIS, OIR and T2DM due to lack of data from the lean group

### Metabolites differentiating OIS from OIR + T2DM

**Fig. 1:**
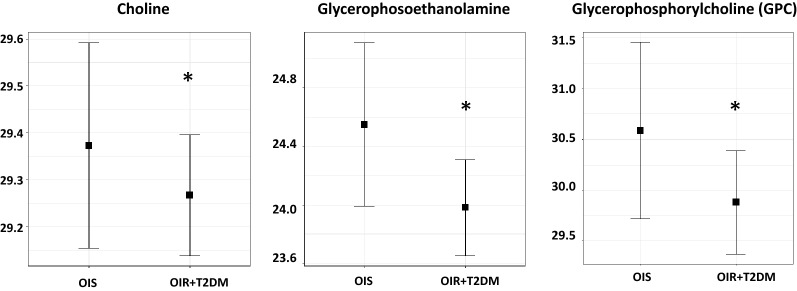
Boxplot of metabolites that belong to the enriched phospholipid pathway differentiating OIS and OIR + T2DM groups. Linear regression was performed to identify significant metabolites differentiating OIS from OIR and T2DM using the R statistical package after correcting for age, gender, BMI and principle components (PC1 and PC2). Y-axis indicates levels of metabolites (log2). *p-value significance level of 0.05 was used

Non-targeted metabolomics of serum samples from the 107 participants was applied to identify metabolites that differentiate OIS vs OIR and OIS vs OIR + T2DM individuals to reveal a metabolic signature of obesity-associated insulin resistance and T2DM. Initial analysis revealed no significant differences in levels of metabolites between OIS and OIR due to their small group sizes (data not shown); however, when combining OIR + T2DM, the linear model revealed 27 metabolites exhibiting significant differences between OIS and OIR + T2DM groups (Table 2). These included metabolites associated with glycolysis, gluconeogenesis and pyruvate metabolism (glucose and 1,5 AG), histidine metabolism (1-methylhistamine, 1-ribosyl-imidazoleacetate and formiminoglutamate) and phospholipid metabolism (choline, glycerophosphoethanolamine and glycerophosphorylcholine). Since the Bonferroni level of significance was not achieved for any of the identified associations, pathway enrichment analysis was performed based on identifying pathways reported by nominally significant metabolites more frequently than can be attributed to random chance. Among the significantly altered metabolic pathways, the phospholipids metabolic pathway was significantly over-represented based on enrichment analysis of the nominally significant metabolites from the group comparisons (p = 3.9E−7). The corresponding metabolites associated with the phospholipids metabolic pathway differentiating OIS from OIR + T2DM included choline, glycerophosphoethanolamine and glycerophosphorylcholine (GPC) (highlighted in Table 2). Figure 1 illustrates levels of significant metabolites that belong to enriched pathways in different study groups. Figure 1 demonstrates higher levels of choline, glycophosphoethanolamine and GPC in OIS compared OIR + T2DM and lean groups. Levels of these metabolites in individual groups are also shown in Additional file 1: Fig. S1.

**Table 2:** Metabolites differentiating OIS from OIR + T2DM

Metabolites	Sub pathway	Super pathway	Fold change	Std. error	p value
1,5-Anhydroglucitol (1,5-Ag)	Glycolysis gluconeogenesis and pyruvate metabolism	Carbohydrate	− 0.92	0.4	0.041
12-Dilinoleoyl-Gpc (18:2/18:2)	Phosphatidylcholine (PC)	Lipid	0.47	0.2	0.037
12-Dilinoleoyl-Gpe (18:2/18:2)*	Phosphatidylethanolamine (PE)	Lipid	1.23	0.51	0.037
1-Methylhistamine	Histidine metabolism	Amino Acid	1.16	0.38	0.007
1-Ribosyl-Imidazoleacetate*	Histidine metabolism	Amino Acid	0.85	0.35	0.03
26-Dihydroxybenzoic Acid	Drug—topical agents	Xenobiotics	1.18	0.42	0.011
3-Amino-2-Piperidone	Urea cycle; arginine and Proline metabolism	Amino Acid	− 0.99	0.37	0.016
5-Methylthioadenosine	Polyamine metabolism	Amino Acid	1.18	0.38	0.006
Alpha-hydroxyisovalerate	Leucine isoleucine and valine metabolism	Amino Acid	0.7	0.27	0.023
Arachidonoylcholine	Fatty acid metabolism (acyl choline)	Lipid	− 1.05	0.41	0.021
*Choline*	*Phospholipid metabolism*	*Lipid*	*− 0.46*	*0.17*	*0.013*
Cortisol	Corticosteroids	Lipid	0.87	0.31	0.012
Docosatrienoate (22:3N3)	Long chain polyunsaturated fatty acid (n3 and n6)	Lipid	0.64	0.25	0.024
Formiminoglutamate	Histidine metabolism	Amino Acid	1.15	0.52	0.05
Gamma-tocopherol/beta-tocopherol	Tocopherol metabolism	Cofactors and Vitamins	1.38	0.55	0.024
Glucose	Glycolysis gluconeogenesis and pyruvate metabolism	Carbohydrate	0.3	0.12	0.025
Glycerol 3-phosphate	Glycerolipid metabolism	Lipid	− 0.93	0.38	0.029
Glycerophosphoethanolamine	Phospholipid metabolism	Lipid	− 1.24	0.47	0.019
Glycerophosphorylcholine (GPC)	Phospholipid metabolism	Lipid	− 1.85	0.77	0.032
HWESASXX*	Tyrosine metabolism	Amino Acid	− 1.44	0.59	0.029
Methionine sulfone	Drug—metabolic	Xenobiotics	0.89	0.34	0.018
Methylphosphate	Benzoate Metabolism	Xenobiotics	− 1.55	0.48	0.006
*N*-Formylanthranilic Acid	Fatty acid metabolism (acyl carnitine monounsaturated)	Lipid	0.9	0.4	0.044
*N*-Stearoyl-Sphinganine (D18:0/18:0)*	Endocannabinoid	Lipid	0.84	0.31	0.015
*N*-Stearoyl-Sphingosine (D18:1/18:0)*	Ceramides	Lipid	0.44	0.2	0.05
Pipecolate	Fatty acid dicarboxylate	Lipid	0.92	0.32	0.011
Ribitol	Vitamin A metabolism	Cofactors and vitamins	0.33	0.14	0.044

Italicized rows represent metabolites that belong to the significantly enriched phospholipids pathway. Linear regression was performed to identify significant metabolites differentiating OIS from OIR and T2DM using the R statistical package after correcting for age, gender, BMI and principle components (PC1 and PC2). A p-value significance level of 0.05 was used. Asterisks (*) on IDs of some metabolites indicate that they have not been officially confirmed based on a standard, but their identities are known with confidence [23]

### Metabolites associated with disease progression

**Fig. 2:**
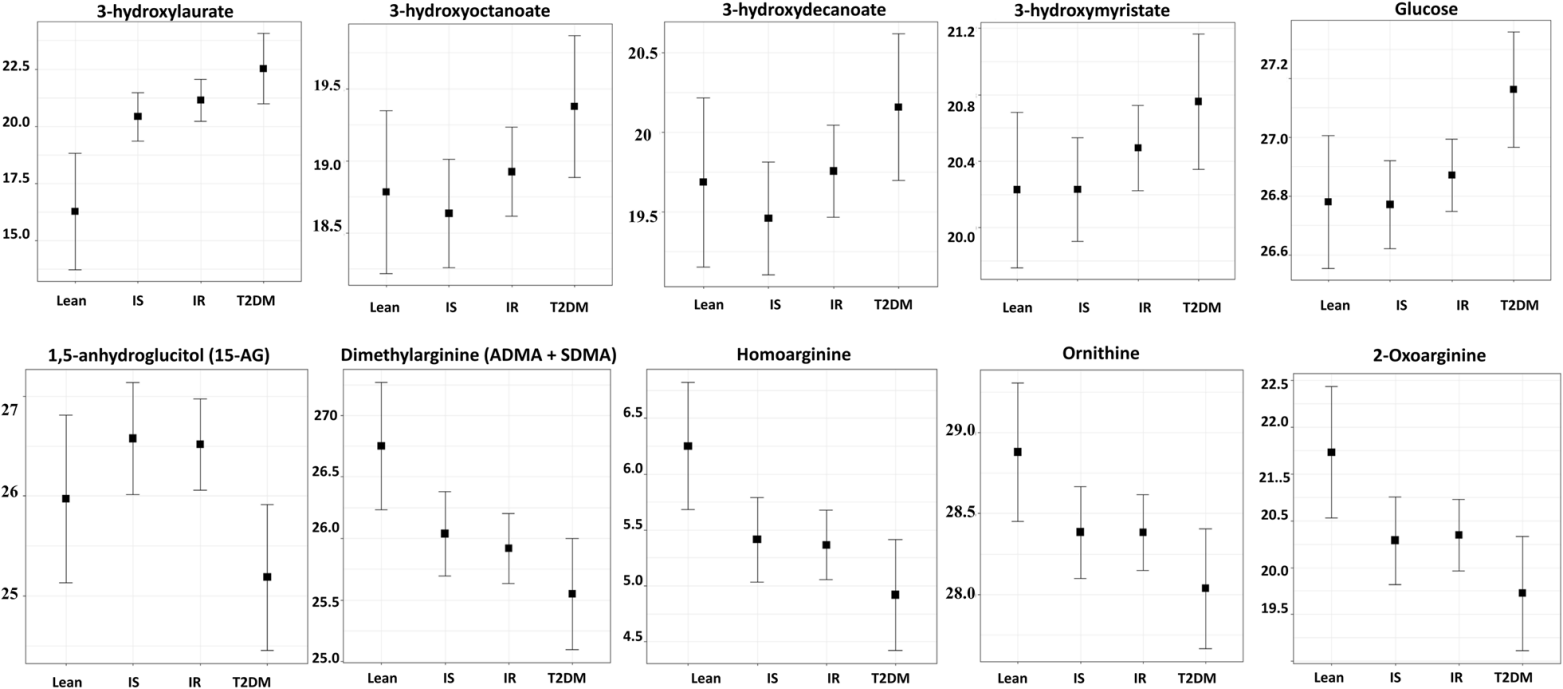
Boxplot of metabolites that belong to the enriched pathways associated with increased risk of obesity-associated insulin resistance and T2DM. Linear regression was performed to identify significant metabolites associated with disease progression using the R statistical package after correcting for age, gender, BMI and principle components (PC1 and PC2). Y-axis indicates levels of metabolites (log2). A p-value significance level of 0.05 was used

An additional a linear model was used to assess the significance of metabolites associated with increased risk of obesity-associated insulin resistance and T2DM (as defined in the method section). Sixty-six metabolites exhibited significant differences with disease progression. The list of metabolites and their associated pathways are shown in Additional file 2: Table S1. These included metabolites associated with glycolysis (glucose), mannose metabolism (mannose), monohydroxy fatty acid (3-hydroxylaurate, 3-hydroxyoctanoate, 3-hydroxydecanoate and 3-hydroxymyristate), medium chain fatty acids (laurate) and urea cycle; arginine and proline metabolism (ADMA + SDMA) among others. Enriched metabolic pathways included glycolysis, gluconeogenesis and the pyruvate metabolic pathway (p = 0.02), fatty acid monohydroxy metabolic pathway, urea cycle metabolic pathway (p = 0.04) and arginine and proline metabolic pathway (p = 0.05). Subsequently, metabolites that showed significant differences with disease progression (Additional file 2: Table S1) within these enriched pathways were identified (Table 3). Figure 2 demonstrates patterns of increased (3-hydroxylaurate, 3-hydrocyoctanoate, 3-hydroxydecanoate, 3-hydroxymyristate, and glucose) or decreased (1,5-AG, ADMA + SDMA, homoarginine, ornithine, 2-oxoarginine) metabolites with disease progression.

**Table 3:** Metabolites that belong to the significantly enriched pathways associated with obesity-associated insulin resistance and T2DM

Metabolites	Sub pathway	Super pathway	Beta value	Std. error	p value
3-Hydroxylaurate	Fatty acid monohydroxy	Lipid	0.3	0.1	0.002
3-Hydroxyoctanoate		Lipid	0.3	0.1	0.008
3-Hydroxydecanoate		Lipid	0.3	0.1	0.009
3-Hydroxymyristate		Lipid	0.2	0.1	0.012
Glucose	Glycolysis gluconeogenesis and pyruvate metabolism	Carbohydrate	0.2	0	0.001
15-Anhydroglucitol (1,5-AG)		Carbohydrate	− 0.4	0.2	0.019
Dimethylarginine (ADMA + SDMA)	Urea cycle; arginine and proline metabolism	Amino Acid	− 0.3	0.1	0.009
Homoarginine		Amino Acid	− 0.3	0.1	0.017
Ornithine		Amino Acid	− 0.2	0.1	0.042
2-Oxoarginine*		Amino acid	− 0.3	0.1	0.045

Linear regression was performed to identify significant metabolites associated with disease progression (lean, OIS, OIR, T2DM) using the R statistical package after correcting for age, gender, BMI and principle components (PC1 and PC2). A p-value significance level of 0.05 was used. Asterisks (*) on IDs of some metabolites indicate that they have not been officially confirmed based on a standard, but their identities are known with confidence [23]

**Fig. 3:**
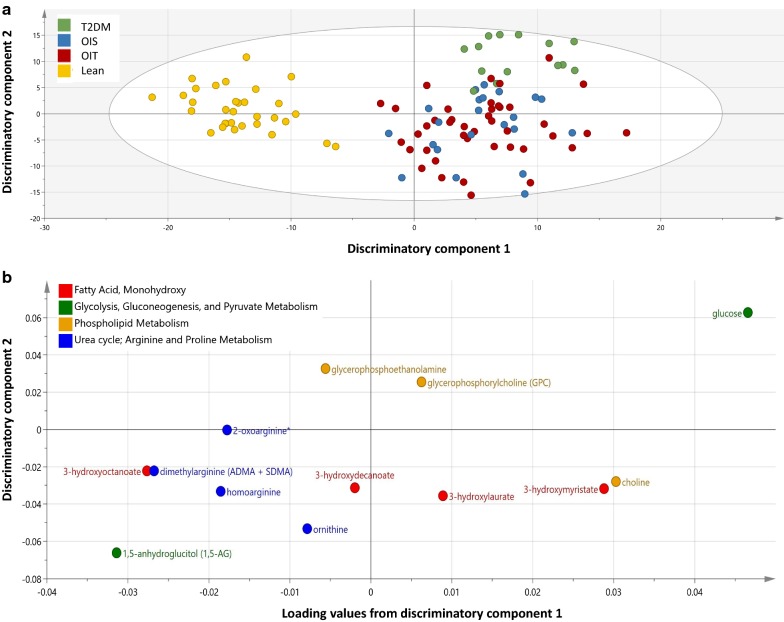
OPLS-DA model comparing metabolites from lean, OIS, OIR and T2DM individuals. **a** A score plot showing the class-discriminatory component 1 (*x*-axis) versus class-discriminatory component 2 (*y*-axis). **b** The corresponding loading plot showing enriched pathways’ associated metabolites differentiating OIS and OIR + T2DM groups or those associated with disease progression

An orthogonal partial least square discriminate analysis (OPLS-DA) comparing subjects from lean, OIS, OIR and T2DM was used for ease of visualization. The model revealed three class-discriminatory components accounting for 48% of the variation in the data due to participant groups (Fig. 3). The score plot in Fig. 3a indicates an x-axis separating the lean group from OIS, OIR and T2DM; the latter group being rather separated along the y-axis. The corresponding loading plot, shown in Fig. 3b, indicates enriched pathways’ associated metabolites significantly differentiating OIS and OIR + T2DM and those associated with disease progression as per linear models. Specifically, higher glucose, choline, GPC, 3-hydroxymyristate and 3-hydroxylaurate and lower 1,5-AG, dimethylarginine (ADMA + SDMA), homoarginine, ornithine and 2-oxoarginine are indicated.

### Correlation of significant metabolites with mediators of metabolic disease

A partial correlation analysis was used to determine traits of disease best associated with metabolites showing significantly differing levels between disease groups. In essence, the correlation between each of such metabolites and each trait was evaluated after correcting for the effect of all other remaining traits. The correlations that remained significant after such correction are listed in Table 4. The trait of liver function enzymes (ALP and ALT), BMI, TAGs, leptin, insulin and HOMA-IR showed the most significant correlations with levels of metabolites differentiating OIS and OIR + T2DM and those associated with disease progression.

**Table 4:** Classical metabolic traits that best predict levels of metabolites differentiating OIS and OIR + T2DM and those associated with disease progression

Metabolite	Partial correlation (R)	Mediator of metabolic disease	p value
3-Hydroxylaurate	0.4	ALP	0.003
3-Hydroxyoctanoate	0.4	ALP	0.002
		BMI	0.003
3-Hydroxydecanoate	0.83	BMI	< 0.001
Glucose	0.4	Insulin	< 0.001
		TAGs	0.001
1,5-Anhydroglucitol (1,5-AG)	− 0.4	Insulin	< 0.001
		TAGs	< 0.001
		HOMA-IR	0.004
Ornithine	0.4	ALT	< 0.001
		Leptin	< 0.001
Choline	0.4	ALT	< 0.001
		Leptin	0.001
Glycerophosphoethanolamine	0.39	BMI	< 0.001

A partial correlation analysis by stepwise linear regression was performed using IBM SPSS version 25, R version 3.2.1. A p-value significance level of 0.005 was used

## Discussion

Obesity triggers a cascade of biochemical changes that increase the risk of various comorbidities including insulin resistance and T2DM. However, some obese individuals seem to be protected against obesity-associated comorbidities. Understanding the underlying mechanisms of this apparently protective phenotype could provide a therapeutic strategy to mitigate the comorbidities associated with pathological obesity. Various studies have investigated the potential mechanisms underlying differences among lean, obese-IS, obese-IR and obese-T2DM individuals [7, 9, 11, 14, 28, 29], however no study has compared the differences in the metabolic signature among these groups as a means to identify potential diagnostic and therapeutic targets. In this study, untargeted metabolomics analysis of serum samples from lean, OIS, OIR and obese-T2DM individuals was utilized to investigate the metabolic pathways underlying progression of insulin resistance and T2DM. Our novel data indicate that the phospholipid metabolites (choline, glycerophosphoethanolamine and glycerophosphorylcholine) were significantly altered when comparing OIS and OIR + T2DM. Additionally, our data confirmed metabolic changes in several metabolic pathways with obesity-associated insulin resistance and T2DM, including fatty acid and arginine metabolism as well as metabolic markers of liver disease, vascular disease, and diabetes. Therefore, the novel metabolites reported here differentiate the metabolically healthy obese group (OIS) from the pathological obese group (OIR + T2DM) and confirm known biomarkers of obesity-associated insulin resistance with potential diagnostic and therapeutic applications. The causative nature of the identified correlations between metabolites and insulin resistance cannot be ruled out particularly as it is recognized that free fatty acids, for instance, increase insulin resistance [30]. Therefore, future in vitro and in vivo functional studies are warranted where the effects of these metabolites on inducing insulin resistance could confirm their functional relevance.

### A novel metabolic signature differentiating OIS and OIR + T2DM

Since there were no difference between OIS and OIR likely due to their small group sizes, the analysis was repeated by comparing OIS and combined OIR and T2DM groups as the latter two groups were matched for obesity and insulin resistance. Three phospholipids were found to differentiate between OIS and OIR + T2DM. These included increased levels of choline and GPC in OIS compared to OIR + T2DM and lean groups, suggesting a protective role in obesity-associated insulin resistance. GPC is a natural precursor of phospholipids and a metabolite derived from phosphatidylcholine. It contributes the most to circulating choline levels; therefore, GPC serves as a precursor for acetylcholine. The latter is an important neurotransmitter and a vasodilator that shows a different microvascular reactivity between IR and IS nondiabetic women [31]. Previous studies have reported that dietary choline levels can also lower the risk of fatty liver disease and liver damage [32]. Glycerophosphoethanolamine was another metabolite that differentiated OIS from OIR + T2DM. Glycerophosphoethanolamine represents a membrane degradation product that has been linked to chronic liver disease [33]. The novel associations between higher levels of these phospholipid metabolites and obesity-associated insulin sensitivity could therefore reflect decreased risk of microvascular disease, small vessel disease, lipotoxic cardiac diseases and non-alcoholic liver disease in the OIS group compared to OIR + T2DM group of participants [34–36].

### Metabolic signature of obesity-associated insulin resistance and T2DM

When comparing the metabolic profiles of lean, obese-IS, IR and T2DM individuals, several metabolites significantly changed with disease progression. These included metabolites that were previously reported in association with insulin resistance and T2DM such as glucose and 1,5-AG [37, 38]. Other identified metabolites were reported in association with comorbidities of insulin resistance and T2DM including fatty acid metabolic disorders (such as 3-hydroxylaurate) [39], impairment of liver function and diabetic status (such as 3-hydroxymyristate and homoarginine) [40, 41] and vascular disease (such as dimethylarginine) [42]. Other novel arginine metabolites were also found to be significantly changed with disease progression including ornithine (a precursor of arginine, also a medication for hepatic encephalopathy) [43] and 2-oxoarginine (a metabolite of arginine catabolism and a marker of argininemia) [44]. Novel metabolites in association with disease progression were also identified including medium chain fatty acids 3-hydroxyoctanoate and 3-hydroxydecanoate that have been reported to be involved in beta-oxidation of longer-chain fatty acids [45, 46]. Previous reports have associated increased plasma levels of 3-hydroxyoctanoate in patients with an inherited deficiency of long-chain 3-hydroxyacyl-CoA dehydrogenase, as a marker of various clinical cases such as recurrent myoglobinuria, hypoketotic hypoglycemic encephalopathy, hypertrophic/dilatative cardiomyopathy, sudden infant death, and fulminant hepatic failure [46, 47].

### Correlation between metabolites differentiating OIS and OIR + T2DM and classical mediators of metabolic disease

When considering correlations between the identified metabolites and classical mediators of metabolic disease such as age, BMI, lipids, FBG, insulin, HOMA-IR and liver function enzymes, a partial correlation analysis revealed several significant associations. Choline, previously shown to be lower in hepatic damage [48], was found to positively correlate with leptin and ALT. Despite its positive correlation with ALT, choline was found to be higher in OIS compared to OIR + T2DM, indicating a relationship between this metabolite and the protective phenotype of OIS individuals that requires further investigation. On the other hand, glycerophosphoethanolamine was found to be associated with BMI, suggesting increased levels of this membrane degradation product with obesity.

### Correlation of disease progression metabolites and classical mediators of metabolic disease

As expected, glucose and 1,5-AG, previously shown to be associated with T2DM, were found to correlate significantly with levels of insulin and circulating triacylglycerol levels. When considering metabolites that were significantly associated with obesity-related comorbidities, a significant correlation between levels of 3-hydroxylaurate and ALP, was revealed. This suggests that 3-hydroxylaurate, a medium chain fatty acid that is associated with intolerance to prolonged fasting and recurrent episodes of hypoglycemic coma, may constitute a novel marker of fatty liver disease. Similarly, 3-hydroxyocanoate was also found to be associated with ALP and BMI, suggesting that it may also be a novel marker of obesity-associated fatty liver disease. 3-hydroxydecanoate was also found to be associated with BMI, suggesting increased levels of another medium-chain fatty acid with a role in the beta-oxidation and obesity. Ornithine, previously shown to be associated with hepatic damage, was found to be associated with leptin and ALT, providing a further evidence of its association with obesity associated non-alcoholic fatty liver disease.

### Study limitations

This has a number of limitations including the relatively low number of participants per group and the cross-sectional nature of the study limited the interpretation of the findings from a pathophysiological point of view. The observational nature of the findings requires functional validation before suggesting any causalities, especially as some findings were based on weak to moderate associations. Furthermore, since blood samples were collected at multiple sites, a batch effect may have occurred, but this was mitigated by standardized protocols for sample collection, processing and storage. It is possible that other unmeasured factors may have impacted our data including dietary habits, medication/supplements and other unknown environmental factors; however, inclusion of principle components in the regression model may have captured part of these potential confounding factors. Finally, controls were not matched for age and gender compared to the study groups, adding an additional variable; however, both age and gender were corrected for in the analysis, but their influence over metabolic differences cannot be ruled out.

## Conclusion

In the comparison between equally obese insulin sensitive and insulin resistance individuals, phospholipid metabolites including choline, glycerophosphoethanolamine and glycerophosphorylcholine (GPC) were significantly altered. In addition, several metabolites were identified and were confirmatory for insulin resistance and T2DM (such as glucose and 1,5-AG) or their comorbidities (such as 3-hydroxylaurate, 3-hydroxymyristate, homoarginine and dimethylarginine). This pilot study also identified novel metabolic markers such as the medium chain fatty acids 3-hydroxyoctanoate and 3-hydroxydecanoate and highlighted their potential link to non-alcoholic fatty liver disease, a hallmark of increased risk of obesity-associated insulin resistance. Further studies are needed to confirm these associations in prospective cohorts and to investigate their functional relevance.

## Supplementary information

**Additional file 1: Figure S1.** Boxplot of metabolites in lean, OIS, OIR and T2DM that belong to the enriched phospholipid pathway differentiating OIS and OIR+T2DM groups.

**Additional file 2: Table S1.** Metabolites associated with disease progression.

## Data Availability

The datasets used and/or analyzed during the current study are available from the corresponding author on reasonable request.

## Abbreviations

BMI: body mass index
DBP: diastolic blood pressure
FBG: fasting blood glucose
HDL: high density lipoprotein
HOMA-IR: homeostatic model assessment
IL-6: interleukin 6
LDL: low density lipoprotein
MAP: mean arterial blood pressure
NARP: non-aqueous reverse phase UHPLC separation
OIS: obese insulin sensitive
OIR: obese insulin resistant
OM: omental
OPLS-DA: orthogonal partial least square discriminate analysis
PCA: principle component analysis
SBP: systolic blood pressure
TAGs: triacylglycerols
T2DM: type 2 diabetes mellitus
UHPLC: ultra-high-performance liquid chromatography
CAD: coronary artery disease
MS: mass spectrometry
1,5-AG: 1,5-anhydroglucitol
UPLC: ultra-performance liquid chromatography
HESI-II: heated electrospray ionization
LC–MS: liquid chromatography–mass spectrometry
HILIC: hydrophilic interaction chromatography
ADMA: asymmetric dimethylarginine
SDMA: symmetric dimethylarginine
