# Supplementary material for: Metabolic signature of obesity-associated insulin resistance and type 2 diabetes
Source: J Transl Med. 2019 Oct 22;17:348. doi: 10.1186/s12967-019-2096-8 (PMC6805293; doi:10.1186/s12967-019-2096-8)

Figure S1. Boxplot of metabolites in lean, OIS, OIR and T2DM that belong to the enriched phospholipid pathway differentiating OIS and OIR+T2DM groups.

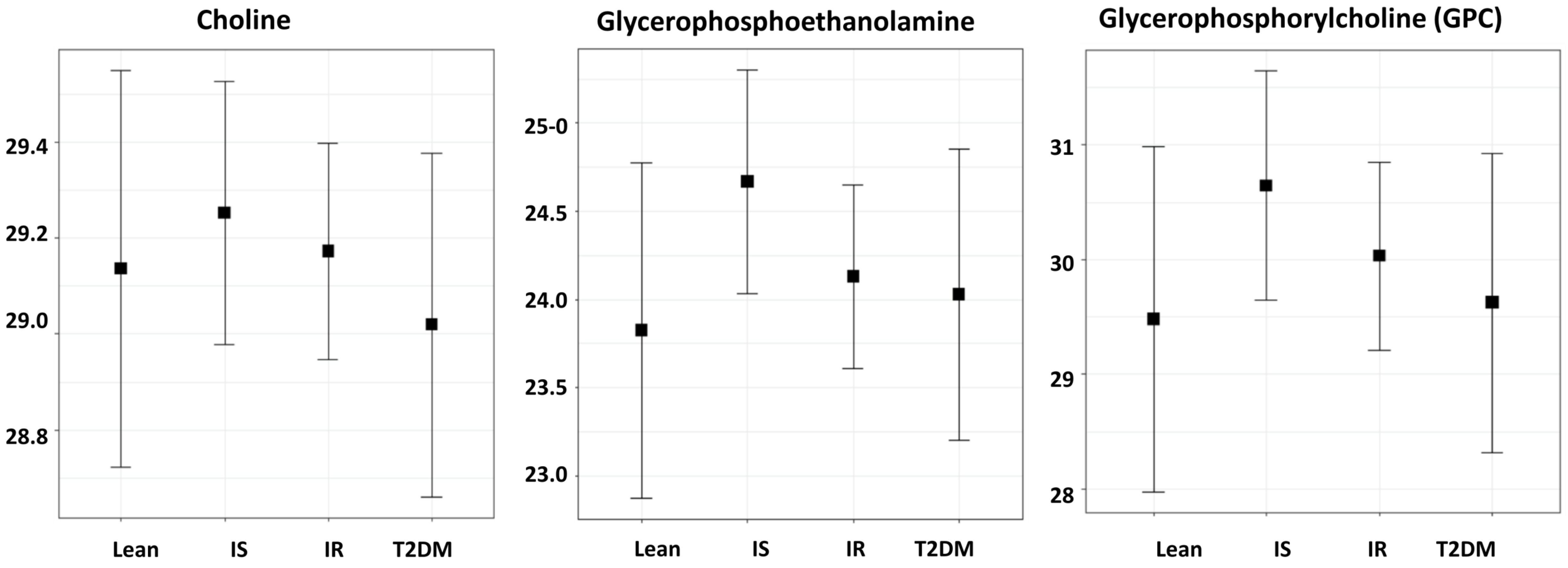

Supplement: Supplementary file 1 — Additional file 1: Figure S1. Boxplot of metabolites in lean, OIS, OIR and T2DM that belong to the enriched phospholipid pathway differentiating OIS and OIR+T2DM groups. [file 12967_2019_2096_MOESM1_ESM.pdf]
