# Supplementary material for: Metabolic signature of obesity-associated insulin resistance and type 2 diabetes
Source: J Transl Med. 2019 Oct 22;17:348. doi: 10.1186/s12967-019-2096-8 (PMC6805293; doi:10.1186/s12967-019-2096-8)
Supplement: Supplementary file 2 — Additional file 2: Table S1. Metabolites associated with disease progression. [file 12967_2019_2096_MOESM2_ESM.docx]

**Table S1. Metabolites associated with disease progression**

| **Metabolites** | **Sub pathway** | **Super pathway** | **Estimate** | **Std. Error** | **t value** | **P value** |
| --- | --- | --- | --- | --- | --- | --- |
| Glucose | Glycolysis Gluconeogenesis and Pyruvate Metabolism | Carbohydrate | 0.15 | 0.04 | 3.4 | 0.001 |
| Palmitoleamide (16:1)* | Fatty Acid Amide | Lipid | -1.97 | 0.00 | -506.8 | 0.001 |
| 3-(2-Hydroxyphenyl)Propionate | Benzoate Metabolism | Xenobiotics | -1.06 | 0.28 | -3.7 | 0.002 |
| Cyclosporin A | Drug - Antiinflammatory Immunosuppressant | Xenobiotics | 1.29 | 0.38 | 3.3 | 0.002 |
| 3-Hydroxylaurate | Fatty Acid Monohydroxy | Lipid | 0.32 | 0.10 | 3.1 | 0.002 |
| Mannose | Fructose Mannose and Galactose Metabolism | Carbohydrate | 0.21 | 0.07 | 3.1 | 0.003 |
| Heme | Hemoglobin and Porphyrin Metabolism | Cofactors and Vitamins | -0.93 | 0.31 | -3.1 | 0.003 |
| N6N6N6-Trimethyllysine | Lysine Metabolism | Amino Acid | -0.44 | 0.15 | -3.0 | 0.003 |
| 2'-Deoxyuridine | Pyrimidine Metabolism Uracil containing | Nucleotide | -0.32 | 0.11 | -2.8 | 0.006 |
| 3-Hydroxyoctanoate | Fatty Acid Monohydroxy | Lipid | 0.30 | 0.11 | 2.7 | 0.008 |
| Glycerophosphoserine* | Phospholipid Metabolism | Lipid | -0.57 | 0.21 | -2.7 | 0.009 |
| Dimethylarginine (Adma + Sdma) | Urea cycle; Arginine and Proline Metabolism | Amino Acid | -0.27 | 0.10 | -2.7 | 0.009 |
| Isoleucylglycine | Dipeptide | Peptide | -0.65 | 0.24 | -2.7 | 0.009 |
| 3-Hydroxydecanoate | Fatty Acid Monohydroxy | Lipid | 0.27 | 0.10 | 2.7 | 0.009 |
| N-Acetylhistidine | Histidine Metabolism | Amino Acid | -0.26 | 0.10 | -2.6 | 0.010 |
| Phosphate | Oxidative Phosphorylation | Energy | 0.25 | 0.10 | 2.6 | 0.011 |
| Laurate (12:0) | Medium Chain Fatty Acid | Lipid | -0.30 | 0.12 | -2.6 | 0.012 |
| 3-Hydroxymyristate | Fatty Acid Monohydroxy | Lipid | 0.23 | 0.09 | 2.6 | 0.012 |
| 26-Dihydroxybenzoic Acid | Drug - Topical Agents | Xenobiotics | 0.41 | 0.16 | 2.6 | 0.012 |
| Tyramine O-Sulfate | Tyrosine Metabolism | Amino Acid | -0.98 | 0.38 | -2.5 | 0.013 |
| Dodecanedioate (C12) | Fatty Acid Dicarboxylate | Lipid | -0.36 | 0.14 | -2.5 | 0.015 |
| 1-Methylhistidine | Histidine Metabolism | Amino Acid | -0.33 | 0.13 | -2.5 | 0.015 |
| Lidocaine | Drug - Analgesics Anesthetics | Xenobiotics | -1.86 | 0.73 | -2.5 | 0.015 |
| Aconitate [Cis Or Trans] | TCA Cycle | Energy | 0.14 | 0.06 | 2.5 | 0.015 |
| Homoarginine | Urea cycle; Arginine and Proline Metabolism | Amino Acid | -0.27 | 0.11 | -2.42 | 0.017 |
| Stearoylcholine* | Fatty Acid Metabolism (Acyl Choline) | Lipid | -0.41 | 0.17 | -2.42 | 0.018 |
| Kynurenine | Tryptophan Metabolism | Amino Acid | -0.19 | 0.08 | -2.40 | 0.019 |
| Spermidine | Polyamine Metabolism | Amino Acid | -0.50 | 0.21 | -2.39 | 0.019 |
| 15-Anhydroglucitol (15-Ag) | Glycolysis Gluconeogenesis and Pyruvate Metabolism | Carbohydrate | -0.41 | 0.17 | -2.38 | 0.019 |
| Tryptophan | Tryptophan Metabolism | Amino Acid | -0.14 | 0.06 | -2.38 | 0.019 |
| N-Acetylhistamine | Histidine Metabolism | Amino Acid | 1.31 | 0.51 | 2.59 | 0.020 |
| Trizma Acetate | Chemical | Xenobiotics | 1.96 | 0.83 | 2.38 | 0.024 |
| 2-Methoxyacetaminophen Glucuronide* | Drug - Analgesics Anesthetics | Xenobiotics | -0.78 | 0.33 | -2.34 | 0.024 |
| Myristoyl-Linoleoyl-Glycerol (14:0/18:2) [1]* | Diacylglycerol | Lipid | -0.35 | 0.15 | -2.29 | 0.025 |
| Docosadioate (C22-Dc) | Fatty Acid Dicarboxylate | Lipid | 0.31 | 0.14 | 2.28 | 0.025 |
| N-Stearoyl-Sphingadienine (D18:2/18:0)* | Ceramides | Lipid | 0.20 | 0.09 | 2.27 | 0.026 |
| N-Stearoylserine* | Endocannabinoid | Lipid | -0.24 | 0.11 | -2.26 | 0.026 |
| Thromboxane B2 | Eicosanoid | Lipid | -2.23 | 0.90 | -2.47 | 0.026 |
| Isoursodeoxycholate | Secondary Bile Acid Metabolism | Lipid | 0.73 | 0.33 | 2.25 | 0.027 |
| Gamma-Tocopherol/Beta-Tocopherol | Tocopherol Metabolism | Cofactors and Vitamins | 0.49 | 0.22 | 2.25 | 0.027 |
| Diacylglycerol (12:0/18:1 14:0/16:1 16:0/14:1) [1]* | Diacylglycerol | Lipid | -0.58 | 0.26 | -2.25 | 0.027 |
| Sphinganine-1-Phosphate | Sphingolipid Synthesis | Lipid | -0.37 | 0.16 | -2.25 | 0.027 |
| Picolinate | Tryptophan Metabolism | Amino Acid | -0.30 | 0.13 | -2.24 | 0.028 |
| Behenoylcarnitine (C22)* | Fatty Acid Metabolism (Acyl Carnitine Long Chain Saturated) | Lipid | -0.31 | 0.14 | -2.21 | 0.032 |
| Linoleoylcholine* | Fatty Acid Metabolism (Acyl Choline) | Lipid | -0.33 | 0.15 | -2.17 | 0.032 |
| Cys-Gly Oxidized | Glutathione Metabolism | Amino Acid | -0.43 | 0.20 | -2.16 | 0.034 |
| Glycochenodeoxycholate Glucuronide (1) | Primary Bile Acid Metabolism | Lipid | -0.57 | 0.27 | -2.13 | 0.036 |
| Adipate | Fatty Acid Dicarboxylate | Lipid | -0.27 | 0.13 | -2.12 | 0.037 |
| 7-Methylguanine | Purine Metabolism Guanine containing | Nucleotide | -0.11 | 0.05 | -2.11 | 0.037 |
| N6N6-Dimethyllysine | Lysine Metabolism | Amino Acid | -0.31 | 0.15 | -2.11 | 0.037 |
| N-Stearoyl-Sphingosine (D18:1/18:0)* | Ceramides | Lipid | 0.17 | 0.08 | 2.10 | 0.038 |
| 1-Linoleoyl-2-Arachidonoyl-Gpe (18:2/20:4)* | Phosphatidylethanolamine (PE) | Lipid | 0.35 | 0.16 | 2.12 | 0.039 |
| Ethylmalonate | Leucine Isoleucine and Valine Metabolism | Amino Acid | 0.26 | 0.13 | 2.10 | 0.039 |
| Butyrate (4:0) | Short Chain Fatty Acid | Lipid | -0.41 | 0.19 | -2.10 | 0.039 |
| Hydroxy-N6N6N6-Trimethyllysine* | Lysine Metabolism | Amino Acid | -0.23 | 0.11 | -2.08 | 0.040 |
| N-Acetylglutamine | Glutamate Metabolism | Amino Acid | -0.26 | 0.12 | -2.07 | 0.041 |
| 1-Palmitoyl-2-Alpha-Linolenoyl-Gpc (16:0/18:3N3)* | Phosphatidylcholine (PC) | Lipid | 0.23 | 0.11 | 2.07 | 0.041 |
| Dihydrocaffeate Sulfate (2) | Food Component/Plant | Xenobiotics | 0.68 | 0.33 | 2.07 | 0.042 |
| Ornithine | Urea cycle; Arginine and Proline Metabolism | Amino Acid | -0.17 | 0.08 | -2.06 | 0.042 |
| Orotidine | Pyrimidine Metabolism Orotate containing | Nucleotide | -0.23 | 0.11 | -2.04 | 0.045 |
| 2-Oxoarginine* | Urea cycle; Arginine and Proline Metabolism | Amino Acid | -0.28 | 0.14 | -2.03 | 0.045 |
| S-Adenosylhomocysteine (Sah) | Methionine Cysteine SAM and Taurine Metabolism | Amino Acid | -0.23 | 0.11 | -2.03 | 0.045 |
| Sulfate* | Chemical | Xenobiotics | 0.10 | 0.05 | 2.02 | 0.046 |
| Quinolinate | Nicotinate and Nicotinamide Metabolism | Cofactors and Vitamins | -0.22 | 0.11 | -2.02 | 0.047 |
| 3-Methoxycatechol Sulfate (1) | Benzoate Metabolism | Xenobiotics | 0.94 | 0.46 | 2.03 | 0.047 |
| 5-Acetylamino-6-Amino-3-Methyluracil | Xanthine Metabolism | Xenobiotics | 0.44 | 0.22 | 2.00 | 0.049 |
